# HIF1 activity in photoreceptors drives type 3 neovascularization and retinal atrophy in a new mouse model of age-related macular degeneration

**DOI:** 10.1038/s41419-025-08028-7

**Published:** 2025-10-06

**Authors:** Fredy Geiger, Thomas Heigl, Luca Merolla, Marcus Yong, Gabriele M. Wögenstein, Larissa P. Govers, Ioanna Tsioti, Antonia Fottner, Marijana Samardzija, Christian Grimm

**Affiliations:** 1 Lab for Retinal Cell Biology, Dept. Ophthalmology, University of Zürich, Zürich, Switzerland; 2 Neuroscience Center, University of Zürich, Zürich, Switzerland

**Keywords:** Retina, Transcriptomics, Apoptosis

## Abstract

Morphological changes in the ageing eye impede oxygen delivery from the choroid to the outer retina causing tissue hypoxia, which activates a molecular response that adapts the transcriptomic fingerprint of the retina and retinal pigment epithelium (RPE). This response, orchestrated by hypoxia-inducible transcription factors (HIFs), leads to the production of pro-angiogenic factors and plays a critical role in the development and pathogenesis of age-related macular degeneration (AMD). To evaluate the specific contribution of HIF1 to this response we expressed a constitutively active form of HIF1A in rod photoreceptors of the adult mouse retina. This elicited a transcriptional response characterized by the upregulation of genes involved in cell death, inflammation and angiogenesis, all of which play an important role in AMD. The HIF1-mediated response in rods caused severe retinal degeneration, disruption of the RPE and retinal neovascularization. Pathological vessels originated from the deep vascular plexus and penetrated the RPE resembling type 3 macular neovascularization observed in over 20% of patients with neovascular AMD. Our study provides further evidence for the involvement of tissue hypoxia in the pathogenesis of AMD and highlights the potential of HIF1A as a therapeutic target.

## Introduction

Age-related macular degeneration (AMD) is the leading cause of blindness in people over the age of 60 in developed countries [1, 2]. With an ageing population, it is estimated that around 288 million people worldwide will be affected by 2040 [1]. Progression of the disease can lead to its late forms that are generally divided into geographic atrophy or dry AMD, and the less common neovascular form or wet AMD (nAMD) [3]. While there is no effective therapy for geographic atrophy, intravitreal injections of anti-vascular endothelial growth factor (VEGF) compounds are the current standard of care for nAMD [4, 5]. However, many patients experience an inadequate response to this medication, due to tolerance, genetic background and/or to the heterogeneity of the neovascular phenotype in nAMD [6, 7]. Recent improvements in in vivo retinal imaging allowed a more detailed characterization of the different neovascular lesions in nAMD and led to a new consensus nomenclature for three subcategories of macular neovascularization (MNV) [8]. While type 1 and type 2 MNV refer to occult and classic choroidal neovascularization respectively, type 3 MNV describes the presence of vessels in the outer retina that originate from the deep retinal capillary plexus. Type 3 MNV replaces previous terms for this form of neovascular AMD, such as ‘deep retinal vascular anomalous complexes’ or ‘retinal angiomatous proliferation’ [9, 10], and was found with a prevalence of 21.4% in nAMD [11].

AMD is a multifactorial disease with age as the major non-modifiable risk factor [12]. Ageing causes various changes in ocular tissues, including thickening of Bruch’s membrane, accumulation of drusen deposits, thinning of the choroid, reduced choroidal blood flow and others [13–16]. These changes impede oxygen delivery from the choroid to the RPE and photoreceptors in the outer retina leading to tissue hypoxia and increased expression of hypoxia-regulated genes through the activity of the hypoxia-inducible transcription factors 1 and 2 (HIF1, HIF2) in the ageing eye [17]. HIFs consist of a constitutively expressed β-subunit (HIFB) and an oxygen-labile α-subunit (HIFA) that is rapidly degraded in normoxia [18, 19]. The normoxic degradation of HIF1A is initiated by the hydroxylation of two prolines (P402 and P564 in humans; P402 and P577 in mice) in the oxygen dependent degradation domain (ODDD) of the protein [20], and mediated by the von Hippel Lindau (VHL) protein complex [21]. To further prevent transcriptional activity of remaining HIF1A proteins in normoxia, factor inhibiting HIF (FIH) hydroxylates the asparagine residue at position 803 (N813 in mice) within the C-terminal transactivation domain (CTAD) [22, 23]. This blocks the interaction with the CBP/p300 coactivators, which are important for full transcriptional activity of HIFs [22, 24]. Hypoxic conditions prevent the hydroxylation of these amino acids enabling HIF1A to complex with the beta subunit and regulate the expression of dozens of genes involved in angiogenesis, cell proliferation, energy metabolism, pH regulation, apoptosis and many others [10, 25]. Activated HIFs not only increase expression of pro-angiogenic factors including VEGFA, angiopoietin 2 (ANG2), fibroblast growth factor 2 (FGF2) and others that are involved in neovascular AMD [26–28], but also modulate cell metabolism and activate apoptotic factors likely contributing to the development of dry AMD as well [29–32]. Therefore, HIFs represent interesting targets for the development of therapies to treat AMD [33].

Several recent studies used mice lacking VHL in rods to investigate the consequences of activated HIF transcription factors. Chronic activity of HIFs in wild type rod photoreceptors resulted in a late onset and slowly progressing retinal degeneration, but no obvious neovascularization [34, 35]. In contrast, activation of HIFs was protective in an acute degenerative model [36] or in genetic models of retinitis pigmentosa [37]. As HIF1 and HIF2 were simultaneously activated in these models, the contribution of each HIF to the phenotypes is unclear. The different observations in these models highlight the intricate nature of HIF transcription factors and stresses the need to study their individual function in more detail and a context-specific manner to fully understand their influence on photoreceptor (patho)physiology. To specifically address the consequences of increased HIF1 activity, we have expressed a normoxia-stable HIF1A protein in adult wild type rod photoreceptors and present a new mouse model with clinical features of MNV type 3 that will be highly useful for studying and identifying mechanisms relevant to nAMD.

## Results

### Normoxia-stable HIF1A is transcriptionally active in normoxic 661W cells

**Fig. 1:**
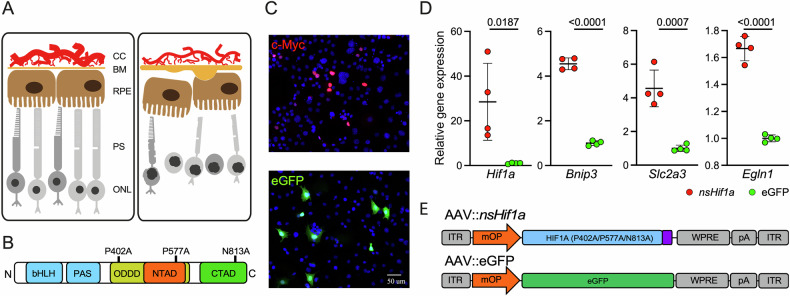
Activity of nsHIF1A in 661 W. **A** Sketch of tissue changes in the old eye leading to hypoxia and degenerative processes. CC choroidal capillaries, BM Bruch’s membrane, RPE retinal pigment epithelium, PS photoreceptor segments, ONL outer nuclear layer. **B** Representation of the domains of HIF1A including the three amino acid changes in nsHIFA1 from mouse. bHLH basic helix-loop-helix, PAS Per-Arnt-Sim domain, ODDD oxygen-dependent degradation domain, NTAD N-terminal transactivation domain, CTAD C-terminal transactivation domain. **C** 661W cells transfected with CMV-*nsHif1a* (top) or CMV-eGFP (bottom) were stained for the Myc-tag (red, top) or imaged for eGFP fluorescence (green, bottom) at 20 h after transfection. Scale bar: 50 µm. **D** Relative gene expression for *Hif1a*, *Bnip2*, *Slc2a3*, and *Egln1* was determined by real-time PCR at 20 h after transfection with CMV-*nsHif1a* (red) or CMV-eGFP (green). Shown are individual data points ±SD. *N* = 4. *p*-values as indicated. Student’s *t* test. **E** Representation of the DNA constructs packaged in AAV2(QuadYF+TV;7m8)/2. DNA elements as labeled. ITR inverted terminal repeat, mOP mouse rhodopsin promoter, WPRE woodchuck hepatitis virus post-transcriptional regulatory element. Violet box: Myc-tag.

Changes in the ageing eye lead to chronic tissue hypoxia in the outer retina, contributing to the development and/or progression of AMD (Fig. 1A). Cells react to hypoxia by inducing a molecular response that is orchestrated by stabilized and activated HIF transcription factors. To mimic this response in normoxic cells, we used a previously described normoxia-stable mouse HIF1A protein (nsHIF1A). This nsHIF1A variant has three key amino acids exchanged in the ODDD (P402A and P577A) and the CTAD (N813A), which prevents degradation and confers transcriptional activity of the protein under normoxic conditions (Fig. 1B) [38].

To evaluate the activity of nsHIF1A in photoreceptor-like cells, we transfected 661 W cells [39] with plasmids expressing either a Myc-tagged nsHIF1A or enhanced green fluorescent protein (eGFP) driven by the ubiquitous CMV promoter. Immunofluorescence for the Myc-tag confirmed the expression and nuclear localization of nsHIF1A in normoxic 661 W cells 20 h after transfection (Fig. 1C). Increased RNA levels for total *Hif1a* (endogenous *Hif1a* plus *nsHif1a*) and the three HIF1 target genes egl-9 family hypoxia inducible factor 1 (*Egln1*), BCL2 interacting protein 3 (*Bnip3*) and solute carrier family 2 member 3 (*Slc2a3*) in *nsHif1a* transfected cells confirmed the activity of the stable HIF1A variant under normoxia (Fig. 1D). In order to investigate the consequences of stabilized and constitutively active HIF1A for rod photoreceptors in vivo, we placed the genes encoding *nsHif1a* and eGFP under the control of the rod-specific mouse opsin (mOP) promoter [40] and packaged the constructs in adeno-associated virus 2 (AAV2) virus particles, resulting in AAV::*nsHif1a* and AAV::eGFP (Fig. 1E).

### Chronic HIF1A activity in rods causes retinal degeneration

**Fig. 2:**
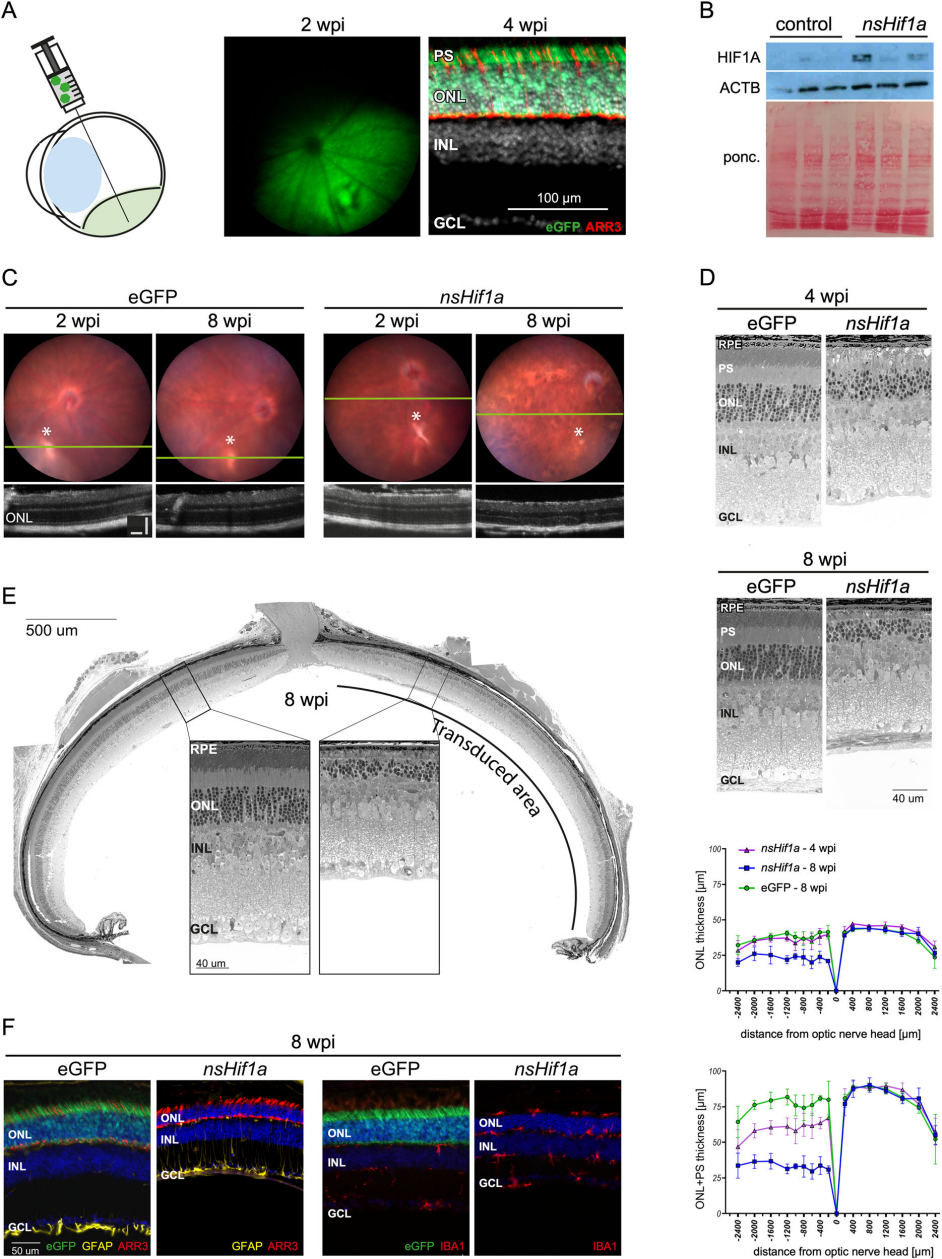
Retinal degeneration induced by chronic HIF1 activity in rods. **A** Sketch of the subretinal AAV application (left). Representative fundus fluorescence image at 2 weeks after AAV-eGFP injection (green, middle) and retinal cross section showing localization of eGFP fluorescence in the photoreceptor layer and lack of co-localization with cone arrestin (ARR3, red, right) at 4 weeks after AAV-eGFP injection. **B** Western blot for HIF1A in retinal samples from mice injected with AAV::eGFP or AAV::*nsHif1a*. Timepoint of analysis: 3 wpi. ACTB and the ponceau (ponc) stained membrane served as loading controls. **C** Fundus images (top) and OCT scans (bottom) at 2 and 8 wpi of mice injected with AAV::*nsHif1a* or AAV::eGFP. Asterisk: injection site. *N* = 6. Scale bar: 100 µm. **D** Retinal morphologies and quantification of the ONL thickness and of the complete photoreceptor layer including outer segments of mice injected with AAV::*nsHif1a* or AAV::eGFP at 4 and 8 wpi as indicated. Shown are spidergrams of means ± SD at indicated distances from the optic nerve head. *N* = 4 per group. **E** Retinal panorama at 8 wpi of a mouse injected with AAV::*nsHif1a*. The transduced area is marked. Insets show magnifications of the boxed areas. Scale bars as indicated. **F** Immunofluorescence of retinal sections at 8 wpi of mice injected with AAV::eGFP or AAV::*nsHif1a*. Green: eGFP. Yellow: GFAP. Red: ARR3 or IBA1. Blue: DAPI. Scale bar: 50 µm. wpi weeks post injection, RPE retinal pigment epithelium, PS photoreceptor segments, ONL outer nuclear layer, INL inner nuclear layer, GCL ganglion cell layer.

AAV::eGFP was injected subretinally to validate the activity and cell type specificity of the promoter used to drive *nsHif1a* expression. At two weeks post injection (wpi), strong eGFP fluorescence covered ~30% of the fundus (Fig. 2A). Retinal sections showed that the eGFP signal was restricted to the outer retina and did not colocalize with cone arrestin (ARR3), indicating that the mOP promoter drove transgene expression specifically in rods (Fig. 2A). eGFP expressed from AAV::eGFP was used as a surrogate marker for the normoxia-stable HIF1A as all attempts to immunolocalize nsHIF1A in the tissue after injection of AAV::*nsHif1a* using anti-Myc or anti-HIF1A antibodies failed to yield reliable signals. However, we detected markedly increased HIF1A in whole retinal extracts from AAV::*nsHif1a* injected mice levels using Western blotting (Fig. 2B, for uncropped original western blots, see ‘Supplemental Material’), although the injected AAVs transduced only about 30% of the retinal area and expression of the mOP-driven transgene was restricted to rods (see above).

Fundus imaging and optical coherence tomography (OCT) scans of AAV::*nsHif1a* injected mice at 2 and 8 wpi showed changes in the pigmentation pattern, less regular retinal layering and thinning of the outer nuclear layer (ONL; Fig. 2C). Light microscopy of retinal sections and quantification of the outer retinal thickness confirmed progressive retinal degeneration with thinning of the outer retina in mice injected with AVV::*nsHifa* but not AAV::eGFP (Fig. 2D). While some photoreceptor segments were still present at 4 wpi, they were almost completely absent by 8 wpi (Fig. 2D). Retinal degeneration in rod-*nsHif1a* mice was restricted to the transduced area (Fig. 2E). The degenerative process was characterized by an activated stress response in Müller glial cells, as indicated by the increased GFAP staining, and by activated microglia cells (IBA1) that invaded the outer retina. However, cones (staining for ARR3) were still present up to 8 wpi (Fig. 2F). By 22 wpi, retinal degeneration had progressed to an advanced stage, and most cones were also lost probably due to secondary degeneration. The inflammatory stress response was still evident with increased GFAP levels in Müller glia cells and activated microglia in the outer retina (Supplementary Fig. S1).

### Chronic HIF1A activity in rods causes expression of angiogenic genes

**Fig. 3:**
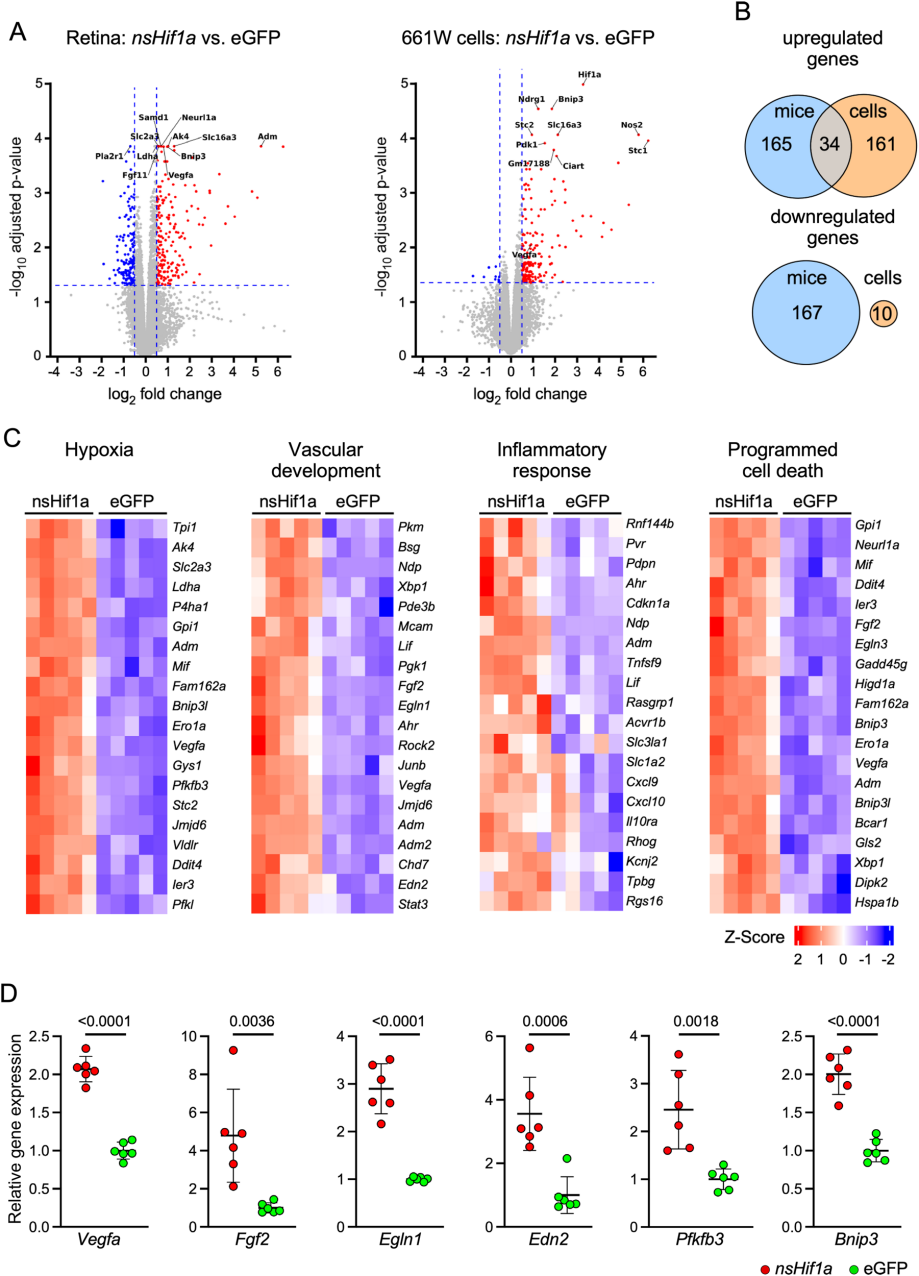
Transcriptomic alterations induced by nsHIF1 in vitro and in vivo. **A** Volcano plots of differentially expressed genes in the retinas of mice injected with AAV::*nsHif1a* (left) or in 661 W cells transfected with *nsHif1a* (right). Timepoints of analysis: 3 wpi for retinas, 20 h for 661 W cells. Thresholds: log_2_FC: 0.5; adjusted *p*-value: 0.05. Red: upregulated genes. Blue: downregulated genes. Gray: genes not significantly regulated. *N* = 5. **B** Venn diagrams of the differentially regulated genes in mice (blue) and 661 W cells (orange). **C** Heat maps of hallmark pathways and biological processes showing the top 20 regulated genes. **D** Real-time PCR of selected genes using retinal RNA from AAV::*nsHif1a* (red) and AAV::eGFP (green) injected mice. Timepoint of analysis: 3 wpi. Shown are individual data points and averages ±SD of *N* = 6. *P*-values as indicated. Student’s *t* test.

To investigate the mechanisms leading to retinal degeneration in rod-*nsHif1a* mice, we analyzed the transcriptomes of the 661 W photoreceptor-like cells transfected with *nsHif1a* in vitro and of rod-*nsHif1a* retinas in vivo at 3 wpi. Volcano plots visualized the differentially expressed genes (Supplementary Tables S1 and S2) and showed that the number of upregulated but not downregulated genes was similar in retinas and cells (Fig. 3A, B). The in vitro and in vivo datasets overlapped by only 34 upregulated genes (Fig. 3B, Supplementary Table S3). Many of the overlapping genes were related to biological processes that involved endoplasmic reticulum stress and protein misfolding in addition to the hypoxic response (Supplementary Fig. S2). This suggests that chronic HIF1 activity induced a severe stress that may have affected protein homeostasis in cells and the retina.

Gene set enrichment analysis of the differentially regulated genes in rod-*nsHif1a* retinas revealed several activated hallmark pathways including ‘hypoxia’, ‘glycolysis’, ‘complement’, ‘inflammatory response’, ‘apoptosis’ and others (Supplementary Table S4). We identified several upregulated biological processes related to cell death, response to oxygen levels, immune response and the vasculature (Supplementary Table S5). Heat maps confirmed the differential regulation of selected pathways and processes (Fig. 3C). Interestingly, most of these pathways and processes are relevant for the development and progression of AMD [41–43]. We validated differential expression of some key genes involved in vascular development and angiogenesis including *Vegfa*, *Fgf2*, *Egln1*, endothelin-2 (*Edn2*) and 6-phosphofructo-2-kinase/fructose-2,6-biphosphatase 3 (*Pfkfb3*), a gene recently associated with pathological ocular angiogenesis [44, 45] (Fig. 3D). The significantly increased expression of all these genes may indicate that chronic nsHIF1A activity in rods affects the homeostasis of the retinal vasculature. *Bnip3*, a known HIF1 target gene, was used as a control for HIF activity (Fig. 3D) [46].

### Chronic HIF1 activity in rods causes RPE disruption

**Fig. 4:**
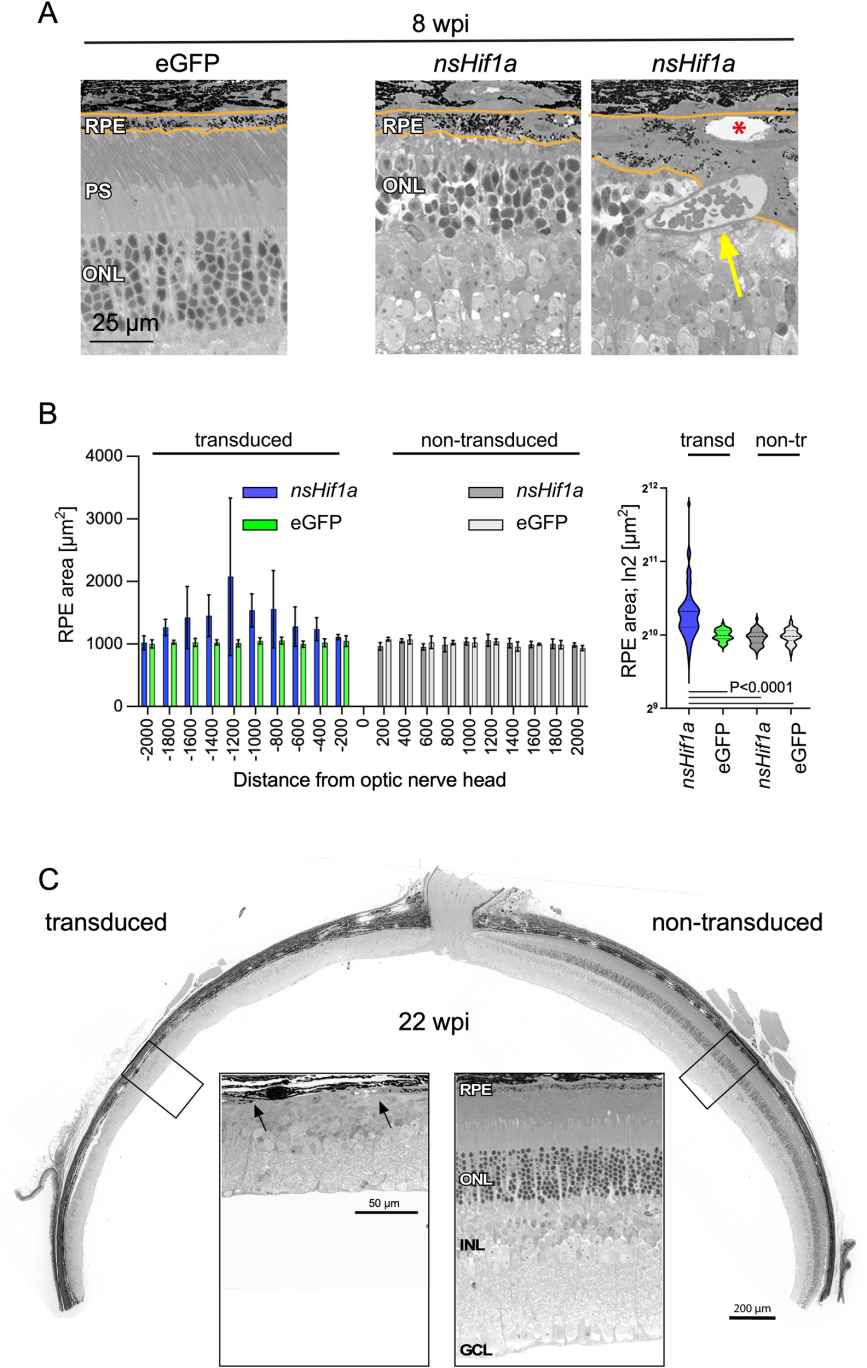
RPE disruption by chronic HIF1 activity in rods. **A** Morphology of the outer retina at 8 wpi of mice injected with AAV::eGFP or AAV::*nsHif1a*, as indicated. Orange lines indicate RPE boundaries. Yellow arrow: blood vessel invading the RPE layer. Red star: large vesicle in the RPE. **B** Quantification of the RPE area on a length of 200 µm on the transduced and non-transduced sides of the optic nerve head. Blue and dark gray bars: quantification of the RPE areas in mice injected with AAV::*nsHif1a*. Green and light gray bars: quantification of the RPE areas in mice injected with AAV::eGFP. Shown are means ± SD of *N* = 4. Violin plots of all data points from the bar graph. Color coding as in the bar graph. *P* value as indicated. One-way ANOVA with Tukey’s multiple comparisons test. **C** Retinal panorama at 22 wpi of a mouse injected with AAV::*nsHif1a*. The transduced and non-transduced areas are indicated. Insets show magnifications of the boxed areas. Black arrows mark a region without RPE. Scale bars: as indicated. RPE retinal pigment epithelium, ONL outer nuclear layer, INL inner nuclear layer, GCL ganglion cell layer. *N* = 3.

Chronic HIF1 activity in rods not only caused retinal degeneration but also affected RPE cell morphology. A marked, irregular thickening and occasional vacuolization of the RPE was observed in the transduced area of rod-*nsHif1a* mice at 8 wpi (Fig. 4A, B). In addition, large vessels were present in the outer retina and even appeared to invade the RPE (Fig. 4A). The increased expression of proangiogenic factors, including *Vegfa, Fgf2* and others (Fig. 3, Supplementary Table S1) may have contributed to this phenotype. With progression of the disease, the RPE deteriorated and disappeared in patches at 22 wpi, resembling geographic atrophy in the transduced but not in the non-transduced area of the retina (Fig. 4C).

### Chronic HIF1 activity in rods causes a phenotype corresponding to MNV type 3

**Fig. 5:**
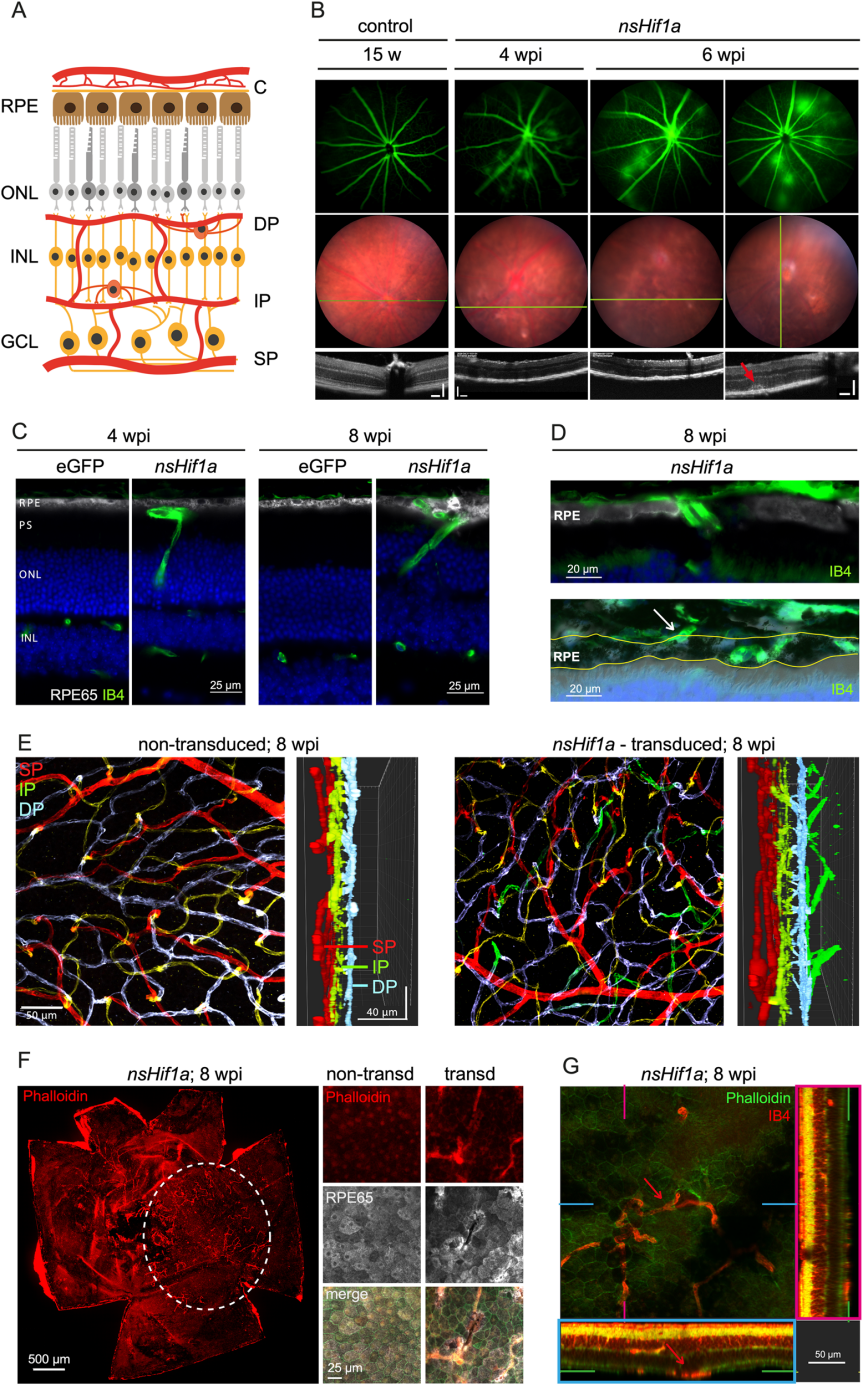
Type 3 neovascularization induced by chronic HIF1 activity in rods. **A** Schematic representation of the nuclear layers and vascular plexi in the mouse eye. RPE retinal pigment epithelium, ONL outer nuclear layer, INL inner nuclear layer, GCL ganglion cell layer, C choroid, DP deep plexus, IP intermediate plexus, SP superficial plexus. **B** Retinal angiography (top), fundus image (middle) and OCT (bottom) of a non-injected wild type mouse at 15 weeks of age and of AAV::*nsHif1a* injected mice at 4 and 6 wpi. Red arrow indicates hyperreflective spot in in the subretinal space. Green lines: position of OCT scans. Scale bars: 100 µm. **C** Immunofluorescence for RPE65 (white) and vessels (green, isolectin-B4) of retinas injected with AAV::eGFP and AAV::*nsHif1a* at 4 and 8 wpi. Scale bar: 25 µm. **D** Higher magnifications of the RPE65 (white) and isolectin-B4 (green) stained retinas of AAV::*nsHif1a* injected mice at 8 wpi. White arrow: blood vessel likely penetrating the Bruch’s membrane. Yellow lines: borders of the RPE. **E** Retinal flat mounts of a non-transduced (left) and an AAV::*nsHif1a* transduced area (right). Flatmounts were stained for vessels (IB4) which were artificially colored in red (superficial plexus), green (intermediate plexus) and blue (deep plexus). Vessels grown into the outer retina are shown in darker green. Side views have been generated from z-stacks. Scale bars: as indicated. **F** RPE flatmount from an AAV::*nsHif1a* injected mouse stained with isolectin-B4 for vessels (red). The white dotted line indicates the transduced area. High-magnification images show the non-transduced (left) or the transduced (right) area that was stained for vessels (IB4, red), the RPE (RPE65, gray) and actin filaments (phalloidin, green). Scale bars: as indicated. **G** Combined RPE/retina flatmount showing an area transduced with AAV::*nsHif1a* at 8 wpi. Vessels (IB4, red), actin filaments (phalloidin, green). Pink and blue lines mark the positions of the side views (denoted by pink and blue boxed areas). Green lines in the side views indicate the position of the RPE. Red arrow: large vessel within the RPE. Scale bar: 50 µm.

Cells in the inner retina receive nourishment from the capillary networks present in the superficial (SP), intermediate (IP), and deep (DP) vascular plexi. Photoreceptors and the RPE are supported by the blood in the vessels of the choroid (C) (Fig. 5A). Fluorescence angiography showed diffuse vascular leakage in the retina of rod-*nsHif1a* mice at 4, 6 and 22 wpi (Fig. 5B, Supplementary Fig. S1) indicating that the vessels detected in the outer retina (Fig. 4) were leaky. Fundus imaging and OCT scans showed pigmentation deficits with pale areas that correlated with hyperreflective OCT spots, indicating irregular morphology and thinning of the outer retina (Fig. 5B). The thinning of the photoreceptor layer was progressive and resulted in a complete loss of the ONL and a partial loss of the RPE at 22 wpi (Supplementary Fig. S1). The vessels detected in the outer retina (Fig. 4) penetrated the ONL as well as the outer segments and occasionally invaded the RPE (Fig. 5C). In rare cases, these vessels appeared to have crossed the Bruch’s membrane (Fig. 5D). Three-dimensional reconstructions of the retinal vasculature in flatmounts from rod-*nsHif1a* mice at 8 wpi suggested that the neovascular vessels originated in the deep capillary plexus and were exclusively found in the transduced retinal region where rods expressed the normoxia-stable HIF1A protein (Fig. 5E).

RPE flatmounts from rod-*nsHif1a* mice further confirmed the presence of vessels within the RPE layer in the transduced nasal region of the eye (Fig. 5F). Phalloidin and RPE65 staining demonstrated that the RPE integrity was disrupted in the transduced area (Fig. 5F) suggesting that the chronic HIF1 activity in rods affected not only the photoreceptor layer but also the adjacent RPE cells. Confocal microscopy of combined retinal and RPE flatmounts stained with phalloidin and isolectin-B4 showed that the vessels that originated from the deep capillary plexus (see above) extended to the subretinal space/RPE layer, forming a new incomplete vascular plexus inside of the RPE layer (Fig. 5G, Supplementary Fig. S1). Collectively, these data suggest that chronic HIF1 activity in rods not only affected photoreceptor survival but also induced formation of new vessel resembling type 3 neovascularization and affected RPE cell integrity.

## Discussion

Angiogenesis, the process of capillary outgrowth from the pre-existing vasculature, is strongly driven by ischemia and hypoxia [47] and is the major complication in nAMD [48]. Here we show that chronic HIF1 activity in healthy adult rods induces the expression of angiogenic factors including VEGFA and FGF2, causes progressive retinal degeneration and RPE disruption, and is sufficient to induce MNV type3-like angiogenesis in the adult mouse retina.

Our data suggest that HIF1 is a major driver of neovascularization in the retina and may be an interesting target for therapy. This is further supported by reports demonstrating a protective effect of genetic inactivation or RNAi-mediated silencing of *Hif1a* in rods in models of chronic hypoxia [34, 49]. Nonetheless, it is important to consider other results that have indicated a protective effect of HIF activation, e.g. in models of NaIO_3_-induced retinal degeneration [36], retinal detachment [50] or retinitis pigmentosa [37] demonstrating that the consequences of HIF1 activity in photoreceptors may depend on the (patho)physiological context of the retina. The loss of photoreceptors in mouse models of RP reduces oxygen consumption in the outer retina resulting in localized tissue hyperoxia, which may further contribute to degeneration [51, 52]. In such a context, HIF1 activation may help to restore a more balanced metabolic state exerting a protective effect on otherwise compromised photoreceptors. We therefore speculate that the shift in cell metabolism induced by HIF1 activation may be beneficial in situations of acute severe oxidative stress or gene mutations that directly affect photoreceptor homeostasis in the absence of hypoxia.

In AMD, however, tissue hypoxia develops slowly over years and HIF transcription factors may be chronically activated leading to photoreceptor stress and cell death. Counteracting HIF activity in this situation may reduce chronic cell stress and support long-term cell survival.

Several animal models with elevated endogenous HIF1 levels have been developed. These models are primarily based on the inactivation of VHL or prolyl hydroxylases (PHDs), which prevents HIF1A degradation under normoxic conditions [35, 53–55]. These models do not show neovascularization when activated in the adult retina. A major difference between these models and the *nsHif1a* mice presented here is the nature of the induced HIF1A protein. The asparagine present in the CTAD of stabilized endogenous HIF1A can be hydroxylated by FIH, reducing its transcriptional activity in normoxia [22, 23] and resulting in only a moderate upregulation of angiogenic factors [56], which may not be sufficient to induce neovascularization. In contrast, the nsHIF1A protein used here carries the N813A mutation in the CTAD, which prevents hydroxylation by FIH making the nsHIF1A protein more transcriptionally active in normoxia. As a result, HIF1 target genes are highly transcribed, leading to the production of high levels of pro-angiogenic factors and other proteins that cause the strong neovascular phenotype observed after delivery of the *nsHif1a* gene to adult normoxic rods.

The phenotype in the rod-*nsHif1a* mice strongly resembles MNV type 3 that is observed in more than 20% [11] of nAMD patients: the new vessels originate in the deep vascular plexus, grow towards the outer retina and invade the RPE. However, and in contrast to MNV3 in AMD patients, the newly formed vessels rarely breached Bruch’s membrane. Since expression of the *nsHif1a* gene was restricted to rod photoreceptors, we speculate that the absence of increased HIF activity in the RPE in our model (but not in AMD eyes) prevented production of factors necessary for vessels to grow further towards the choroid.

Several mouse models have been published that show abnormal vessel growth in the retina. Many of these models are related to the hypoxic response and are based on the genetic inactivation of *Vhl* in several retinal cell types in addition to photoreceptors, or on the overexpression of *Vegfa* [57]. However, the genetic modifications in these models are already active early after birth and therefore interfere with postnatal retinal development, including the formation of the three vascular plexi in the retina. The resulting early pathology is therefore mixed with developmental processes and may not accurately represent the mechanisms causing neovascularization in the aged eyes of AMD patients. By expressing *nsHif1a* in rods of the adult retina, we have avoided this complication and present a model that allows for the specific analysis of neovascular processes in the adult retina and the testing of therapeutic interventions. Furthermore, the slow degeneration of the retina in *rod*^*ΔVhl*^ mice may be caused by a metabolic shift in photoreceptors [58], while the fast degeneration in rod-*nsHif1a* mice appears to be mainly driven by neovascularization. The pathologies in the two mouse models resemble the slowly developing geographic atrophy and the fast-progressing degeneration in neovascular AMD. Because both pathologies are driven by HIF transcription factors, the data suggest that therapies targeting HIF activity early in disease development or progression may be beneficial for patients suffering from either form of AMD.

## Materials and methods

### Mice

Wild-type 129S6 mice were tested by PCR to be free of the rd8 mutation and maintained as breeding colonies at the Laboratory Animal Service Center (LASC) of the University Zürich under a 14/10-hour light-dark cycle with an average light intensity of 60-150 lux at cage level. Mice had access to food and water *ad libitum*. For tissue sample preparations, mice were euthanized using CO_2_ followed by decapitation.

### Ethics

All experimental procedures were conducted in accordance with “The Association for Research in Vision and Ophthalmology” statement on animal use in ophthalmic and vision research, as well as the regulations of the ethics committee of the veterinary authorities of the Kanton Zurich, Switzerland (license number: ZH105/2022).

### Cloning and virus production

The pcDNA3 *mHif-1a* MYC (P402A/P577A/N813A) plasmid encoding the normoxia-stable HIF1A (*nsHif1a*) with a Myc-tag was a gift from Celeste Simon (Addgene plasmid # 44028) [38]. The *nsHif1a* sequence was cloned into an AAV plasmid backbone containing mOP, a mouse rhodopsin promoter (a gift from Sanford L. Boye) [40]. The control virus was generated using the sequence for enhanced eGFP in place of the *nsHif1a* DNA sequence. All AAVs were produced by the Viral Vector Facility of the Neuroscience Center Zurich. The mOP-*nsHif1a* and mOP-eGFP DNA constructs were packaged in the AAV2(QuadYF+TV;7m8)/2 capsid [59]. To test the *nsHif1a* gene in cells we exchanged the mOP promoter with a human cytomegalovirus (CMV) immediate early enhancer and promoter and used a pcDNA CMV-eGFP plasmid for control transfections.

### Cell culture and transfection

Mouse retinoblastoma-derived 661 W cells [39] were cultured in 100 mm cell culture dishes using Dulbecco’s Modified Eagle Medium (DMEM) (Gibco, LifeTechnologies, Zug, Switzerland) supplemented with 10% fetal bovine serum (Gibco), and 10,000 U/ml penicillin-streptomycin (Gibco). The cells were maintained in a sterile, humidified environment at 37 °C in 21% O_2_ and 5% CO_2_. Transfections were performed using 1 µg plasmid and the lipofectamine 3000 transfection reagent (Thermo Fisher Scientific, Waltham, MA USA). Cells were harvested 20 h after transfection for RNA isolation or fixed for immunofluorescence.

### Subretinal AAV injections

Injections were performed between postnatal days 29–32 as described [60]. Briefly, the pupils of the mice were dilated with Mydriaticum Dispersa 0.5% (Omni Vision AG, Neuhausen am Rheinfall, Switzerland) and Neosynephrin 5% (Ursapharm Schweiz GmbH, Hünenberg, Switzerland). Animals were anesthetized with a subcutaneous injection of ketamine (85 mg/kg; Parke-Davis, Berlin, Germany) and xylazine (10 mg/kg; Bayer AG, Leverkusen, Germany). Lacrinorm (Bausch & Lomb Swiss AG, Zug, Switzerland) was applied to keep the eyes moist during the procedure. The mice were placed on a heating pad set to 37 °C and their heads were stabilized using a stereotactic adapter (Hugo Sachs Elektronik - Harvard Apparatus GmbH, March-Hugstetten, Germany) [61]. After puncturing the temporal sclera just below the ora serata with a 30 G needle, a total of 1 × 109 viral genomes in 1 µl volume was injected subretinally using a 5 µl Hamilton syringe (Hamilton Bonaduz AG, Bonaduz, Switzerland), mounted on a micromanipulator (H. Saur Laborbedarf, Reutlingen, Germany). To facilitate visualization and control during the injections, fluorescein (1 mg/mL, Akorn Inc., IL, USA) was added to the injection solution at a concentration of 10%. After the injections, anesthesia was reversed with atipazemol (2 mg/kg; Graeub, Bern, Switzerland) and the mice were kept on a heating pad until fully awake. Injections were checked after two weeks by fundus imaging and OCT scans using the Micron IV system (Phoenix Research Labs, Pleasanton, CA, USA, see below). Eyes with injection-related complications such as hemorrhages, retinal degeneration or persistent retinal detachment were excluded from the study at this time point. No randomization was used.

### Fundus imaging, OCT and retinal angiography

Pupil dilation was performed as described above. The subcutaneously injected anesthetic solution used for the imaging procedure included acepromazine (3 mg/kg, Fatro S.p.A., Ozzano Emilia, Italy) in addition to ketamine and xylazine. A drop of 2% Methocel (Omni Vision AG, Neuhausen am Rheinfall, Switzerland) was applied to keep the eyes moist during the procedure. For angiography, 50 µl of 2% fluorescein (0.1 mg/mL, Akorn Inc., IL, USA) was injected intraperitoneally. Fundus images, fluorescein fundus images and OCT scans were acquired using the Micron IV system (Phoenix) as previously described [62].

### Retinal morphology

Eyes were marked dorsally, enucleated, and fixed in glutaraldehyde (2.5% in cacodylate buffer) for 12–16 h at 4 °C. Fixed eyes were trimmed, postfixed in 1% osmium tetroxide, and embedded in EMbed 812 (Biolyst Scientific, Morgantown, US) as described previously [63]. Retinal cross sections of 0.5 µm thickness were cut through the optic nerve head, stained with toluidine blue, and analyzed by light microscopy (AxioImager Z2, Carls Zeiss AG, Feldbach, Switzerland). Photoreceptor segment lengths and ONL thickness were measured on reconstructed retinal panoramas at defined positions from the optic nerve head using the ruler tool in Adobe Photoshop CS6 (Adobe Systems, Inc.). To quantify RPE areas, measurements (in µm^2^) were taken at 200 µm intervals along a 200 µm length using the freehand area selection tool in ImageJ (version 1.54f). For the Student’s *t* test, all datapoints from the transduced areas of all mice injected with *nsHif1a* or eGFP were used.

### Immunofluorescence

Eyes were marked at the dorsal limbus, enucleated, and fixed in 4% paraformaldehyde (PFA) in PBS for 10 min. After puncturing the cornea with a 21 G needle, the eyes were further fixed in PFA at 4 °C for 1.5 to 2 h. Subsequently, the cornea and lens were removed, and the eyeballs fixed in PFA for an additional 10 min. The fixed eyes were cryoprotected in 30% sucrose for 2 h at 4 °C and embedded in tissue freezing medium (Leica Biosystems, 81-0771-00). 12-µm thick sections were cut using a Leica cryostat (Biosystems Switzerland AG, CM1860). Immunolabelling was performed with primary antibodies (Table 1) that were incubated with the sections overnight at 4 °C in a blocking solution containing 3% normal goat serum (Sigma-Aldrich) and 0.3% Triton X-100 (Sigma-Aldrich) in phosphate buffer. Sections were washed three times with PBS and incubated with appropriate secondary antibodies in blocking solution for 1 h at room temperature. Sections were washed once with PBS, counterstained with DAPI and after two additional washing steps, slides were mounted using Mowiol (Carl Roth GmbH, Karlsruhe, Germany) and imaged (AxioImager Z2, Carls Zeiss AG).

**Table 1:** Primary antibodies and IB4.

Antibody	Cat. number	Vendor	Application	Dilution
Myc-Tag	#2276	Cell Signaling	Immunofluorescence	1:1000
ARR3	AB15282	Merck Millipore Chemicals	Immunofluorescence	1:500
GFAP	G3893	Sigma-Aldrich	Immunofluorescence	1:250
IB4	I21413	Invitrogen	Immunofluorescence	1:100
RPE65	Pin-5; custom made	Pineda	Immunofluorescence	1:500
Phalloidin	A12379	Invitrogen	Immunofluorescence	1:200
IBA1	019-19741	Wako	Immunofluorescence	1:500
RHO	O4886	Sigma-Aldrich	Immunofluorescence	1:200
HIF1A	NB100-479	Novus Biologicals	Western blotting	1:1000
ACTB	A5316	Sigma-Aldrich	Western blotting	1:10000

### Whole mounts

Whole mounts of the RPE with or without the retina were performed as previously described [64]. Briefly, eyes were marked dorsally, isolated, and incubated in 2% PFA in PBS for 10 min. After puncturing the cornea with a 21 G needle, the eyes were further fixed in 2% PFA for 20 min before being transferred to PBS. The cornea and lens were removed, and the eyes were dissected and cut into cloverleaf structures in PBS. Depending on the type of whole mount preparation, the retina was or was not separated from the RPE. The whole mounts were further fixed in 4% PFA in PBS for 1 h, then blocked for 2 h in a blocking solution containing 3% NGS, 0.3% Triton X-100 in PBS. Whole mounts were incubated overnight with primary antibodies (Table 1) in blocking solution. After washing, the samples were incubated with appropriate secondary antibodies for 2 h, washed, mounted on glass slides, and prepared for analysis. Whole mounts were analyzed using a confocal microscope (Axio Observer.Z1/7 Carls Zeiss AG, Feldbach, Switzerland). The different vascular plexi were manually color coded in ImageJ (version 1.54 f) and 3D reconstructions and sectional images were generated using Imaris Viewer (version 10.0.0, Oxford Instruments, Abingdon, UK).

### RNA isolation, cDNA synthesis and semi-quantitative real-time PCR

Retinas were isolated through a corneal incision and snap-frozen in liquid nitrogen. RNA isolation, cDNA synthesis and semiquantitative real-time PCR were performed as described [65]. Briefly, RNA was isolated from tissues or cells using an RNA isolation kit (NucleoSpin RNA, Macherey-Nagel GmbH & co.KG, Düren, Germany). First-strand cDNA was synthesized using M-MLV reverse transcriptase (Promega, Dübendorf, Switzerland), oligo-dT primers and 1 μg of total RNA. Gene expression was analyzed by semiquantitative real-time PCR (QuantStudio 3, Thermo Fisher Scientific, MA, USA) using 10 ng of cDNA template and PowerUp SYBR green master mix (Thermo Fisher Scientific). Primers (Table 2) were designed to span large intronic regions and avoid known SNPs in the mouse sequence. β-Actin (*Actb*) was used as housekeeping gene for normalization and relative expressions were calculated using the comparative threshold cycle method (2^-ΔΔCT^) [66]. Expression levels were tested for each gene individually using Student’s *t* tests.

**Table 2:** Primer sequences for real-time PCR.

Gene	Forward primer (5’-3’)	Reverse primer (5’-3’)
*Actb*	CAACGGCTCCGGCATGTGC	CTCTTGCTCTGGGCCTCG
*Bnip3*	CCTGTCGCAGTTGGGTTC	GAAGTGCAGTTCTACCCAGGAG
*Edn2*	AGACCTCCTCCGAAAGCTG	CTGGCTGTAGCTGGCAAAG
*Egln1*	GCAGCATGGACGACCTGAT	CAACGTGACGGACATAGCCT
*Fgf2*	TGTGTCTATCAAGGGAGTGTGTGC	ACCAACTGGAGTATTTCCGTGACCG
*Hif1a*	GTCTCCTTTACCTTCATCGGA	GGATTCTTTGCCTCTGTGTCT
*Pfkfb3*	TCGCCGAATACAGCTACGAA	AGCGAGTCAGCTTCTTGGAG
*Slc2a3*	AGGTCACCCAACTACGTCCA	CACCCGCGTCCTTGAAGATT
*Vegfa*	ACTTGTGTTGGGAGGAGGATGTC	AATGGGTTTGTCGTGTTTCTGG

### Transcriptomics and data analysis

mRNA from 661 W cells and murine retina was prepared using Illumina Stranded mRNA Prep to generate antisense paired-end reads. Sequencing was performed on an Illumina NovaSeq X Plus (Illumina, Eindhoven, Netherlands) at the Functional Genomics Center Zurich (FGCZ, University of Zurich, Zurich, Switzerland). Data were processed in R (v4.4.3) as follows: a custom index of the mouse reference genome (GRCm39, primary assembly only) was built using Rsubread (v2.18.0), and reads were aligned with align() using gencode M36 basic annotation. Gene-level quantification was performed with featureCounts() (Rsubread), using antisense strand-specific mode and the same GTF annotation.

Genes with low expression were filtered using filterByExpr() in edgeR (v4.2.2), with a minimum count threshold derived from the library size. Normalization was performed with normLibSizes(), and expression values were transformed using voomWithQualityWeights() from limma (v3.60.6). Linear modeling was done with lmFit() and contrasts defined to compare data. Differential expression was determined using an adjusted *p*-value (FDR) of <0.05 and a |log₂FC| > 0.5. Gene set enrichment analysis was performed using fgsea (v1.30.0) with mouse-specific gene sets from MSigDB v7.5.1 (Hallmark, GO BP, GO MF, GO CC, KEGG). Genes were ranked by moderated *t*-statistics. Visualization was done with ggplot2 (v3.5.1), ComplexHeatmap (v2.20.0), and VennDiagram (v1.7.3).

### Protein isolation and Western blotting

Isolated retinas were transferred into 200 μl Tris-HCl (100 mM, pH 8.2, 4 °C) and homogenized by sonication (10 pulses of 0.3 s and 30% amplitude) with an ultrasonic homogenizer (Branson 450 Digital Sonifier). Samples were centrifuged at 1000 relative centrifugal force (RCF) for 10 min at 4 °C and the supernatant was transferred into a new tube. Protein concentrations were measured with the Pierce BCA Protein Assay Kit (Pierce^TM^, Thermo Scientific) according to manufacturer’s instructions. Protein samples were stored at −20 °C until further use. The protein homogenate was mixed with 1/3 volume of 4x sample buffer (250 mM 1 M Tris/HCl (pH 6.8), 20% glycerol, 4.6% SDS, 0.04% bromophenol blue, 10% 2-mercaptoethanol) and heated at 85 °C for 10 min. 20 μg of protein were run on a 10% SDS PAGE and blotted onto a nitrocellulose membrane using the Trans-Blot Turbo Transfer System (Bio-Rad Laboratories, Hercules, CA, USA). The membrane was blocked in 5% nonfat dry milk (Bio-Rad) in TBST (Tris-Buffered Saline with 0.1% Tween) for 1 h at RT and incubated with the primary antibody for 36 h at 4 °C. Following three washing steps with TBST, the membrane was incubated with the secondary antibody for 1 h at RT. Membranes were washed with TBST and secondary antibodies were detected by chemiluminescence (PerkinElmer, Waltham, MA, USA) using X-ray films (Fujifilm, Minato, Japan).

## Supplementary information

Table S1

Table S2

Table S3

Table S4

Table S5

Figure S1

Figure S2

Supplemental figure legends

Supplemental file 1

Supplementary information

## Data Availability

Transcriptomic data are deposited in GEO and are accessible through the GEO Series accession number GSE296005. R-scripts and codes are available from the authors on request.
