# Supplementary figures and images for: HIF1 activity in photoreceptors drives type 3 neovascularization and retinal atrophy in a new mouse model of age-related macular degeneration
Source: Cell Death Dis. 2025 Oct 6;16(1):687. doi: 10.1038/s41419-025-08028-7 (PMC12500916; doi:10.1038/s41419-025-08028-7)

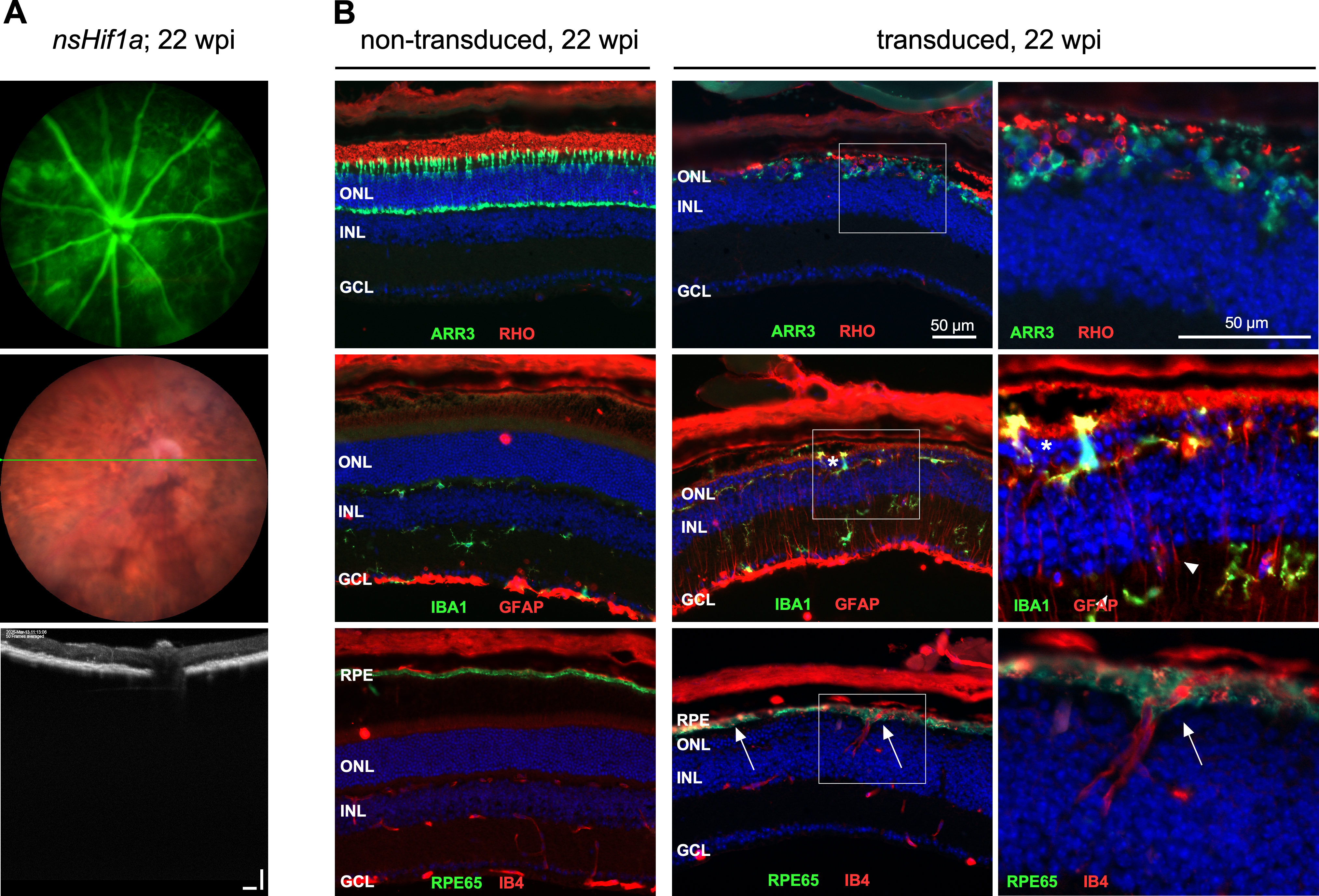

Supplement: Supplementary file 6 — Figure S1 [file 41419_2025_8028_MOESM6_ESM.png]

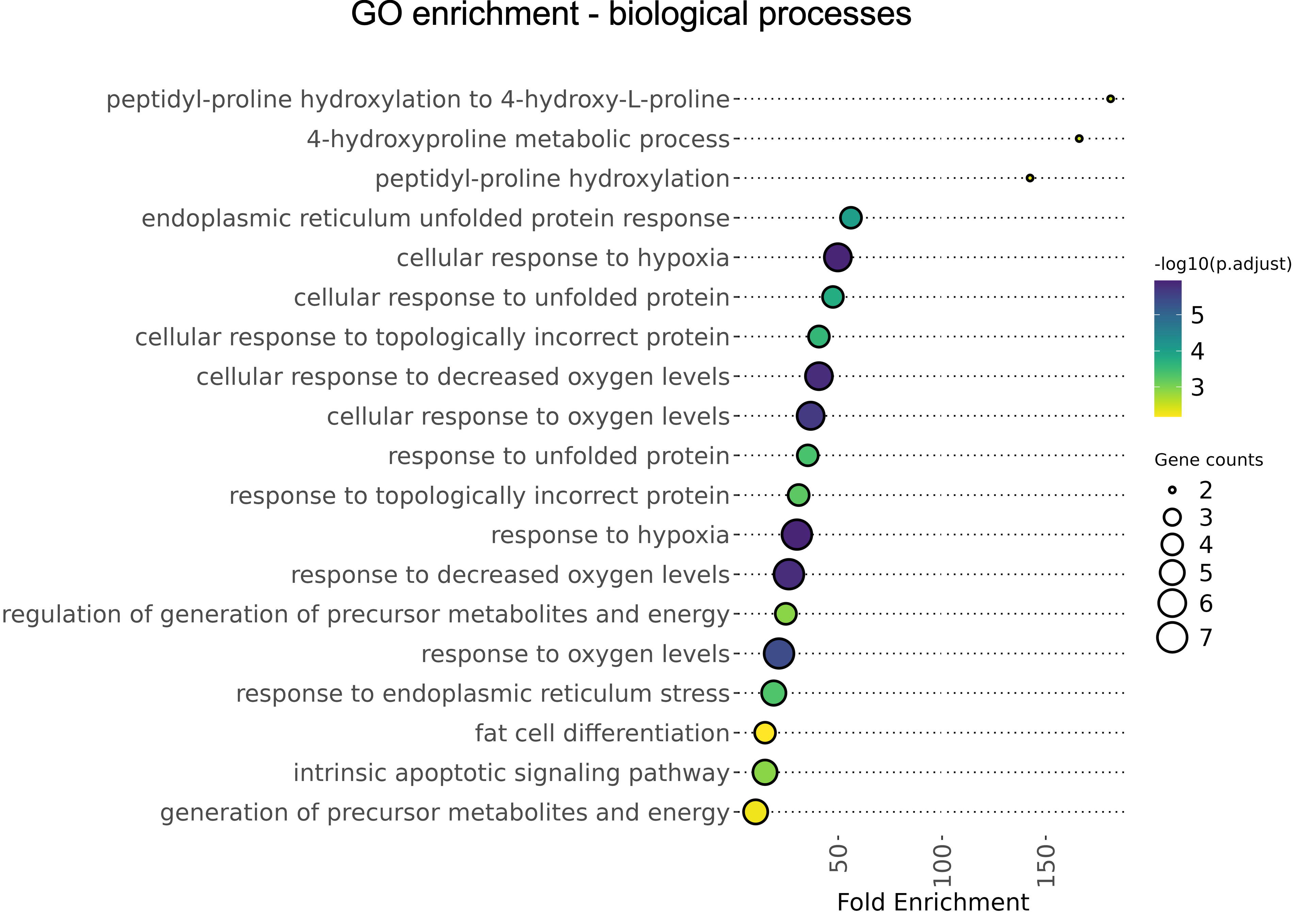

Supplement: Supplementary file 7 — Figure S2 [file 41419_2025_8028_MOESM7_ESM.png]

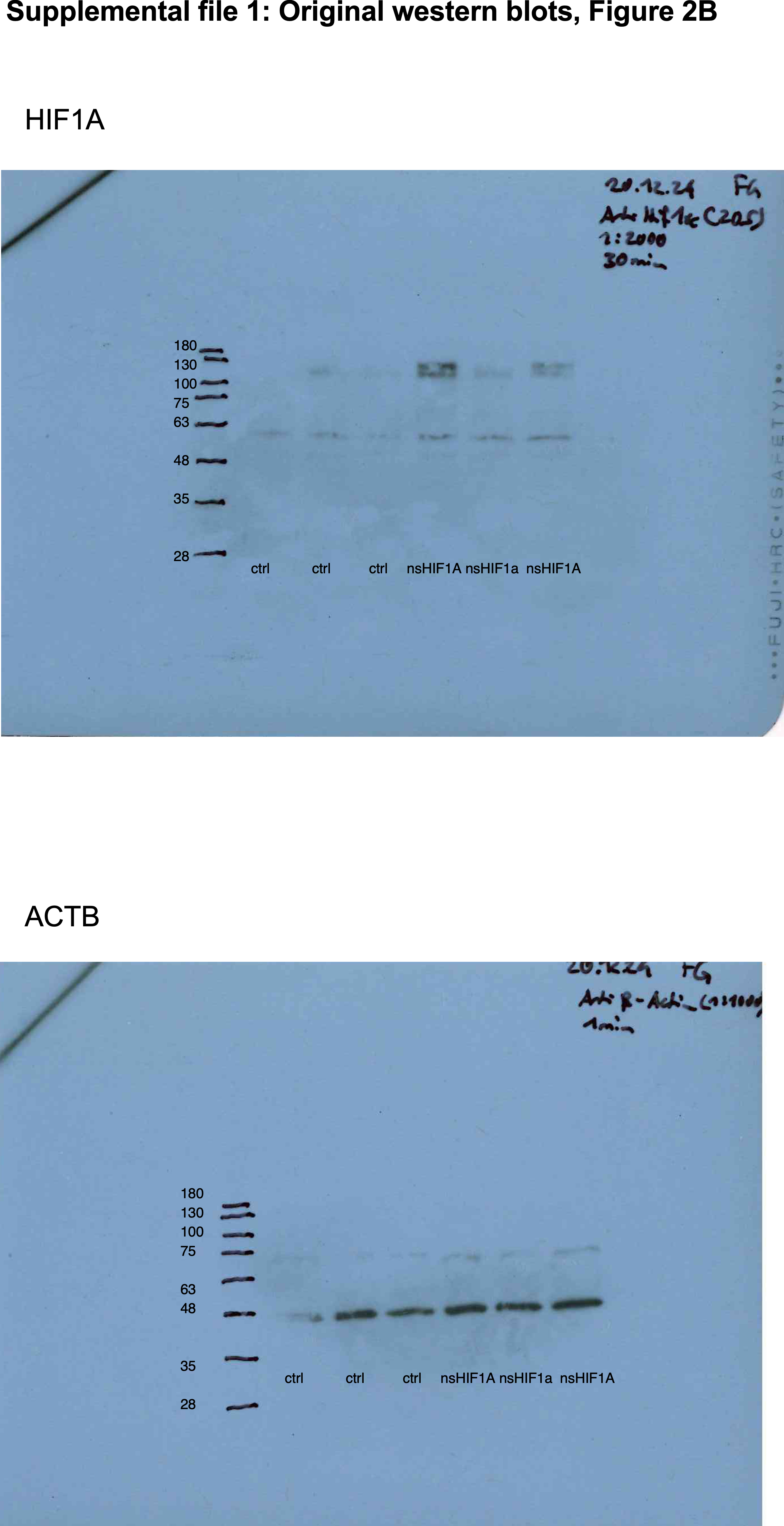

Supplement: Supplementary file 9 — Supplemental file 1 [file 41419_2025_8028_MOESM9_ESM.png]
