# Supplementary material for: HIF1 activity in photoreceptors drives type 3 neovascularization and retinal atrophy in a new mouse model of age-related macular degeneration
Source: Cell Death Dis. 2025 Oct 6;16(1):687. doi: 10.1038/s41419-025-08028-7 (PMC12500916; doi:10.1038/s41419-025-08028-7)
Supplement: Supplementary file 8 — Supplemental figure legends [file 41419_2025_8028_MOESM8_ESM.docx]

**Supplemental figure legends**

**Figure S1: Retinal phenotype of *nsHif1a* mice at 22 wpi. (A)** Retinal angiography (top), fundus image (middle) and OCT (bottom) of an AAV::*nsHif1a* injected mouse at 22 wpi. Green line: position of the OCT scan. Scale bars: 100 µm. **(B)** Immunofluorescence of retinal sections at 22 wpi of a mouse injected with AAV::*nsHif1a*. Shown are the non-transduced and the transduced areas. Top row: ARR3 (green) and RHO (red); middle row: IBA1 (green) and GFAP (red); bottom row: RPE65 (green) and IB4 (red). Blue: DAPI. Magnifications of boxed areas are shown on the right. *: activated microglia. White arrows: vessels within the RPE. Arrowheads: GFAP in activated Müller glia. Scale bars: 50 µm.

**Figure S2: Biological processes enrichment analysis in cells and retina expressing *nsHif1a*.** GO enrichment analysis of the 34 genes found upregulated in both *nsHif1a*-transfected 661W cells and retinas injected with AAV::*nsHif1a*.
