# Supplementary material for: HIF1 activity in photoreceptors drives type 3 neovascularization and retinal atrophy in a new mouse model of age-related macular degeneration
Source: Cell Death Dis. 2025 Oct 6;16(1):687. doi: 10.1038/s41419-025-08028-7 (PMC12500916; doi:10.1038/s41419-025-08028-7)
Supplement: Supplementary file 10 — Supplementary information [file 41419_2025_8028_MOESM10_ESM.docx]

**SUPPLEMENTARY INFORMATION**

**Figure S1: File type: png**

Retinal angiography, fundus image, OCT and morphology of *nsHif1a* mice at 22 wpi show vascular leakage, retinal degeneration with the loss of photoreceptors, and neovascular vessels infiltrating the RPE.

**Figure S2: File type: png**

Biological processes enrichment analysis in cells and retina expressing *nsHif1a*. GO enrichment analysis of the 34 genes found upregulated in both *nsHif1a*-transfected 661W cells and retinas injected with AAV::*nsHif1a*.

**Table S1: File type: xlsx**

Differentially upregulated genes in rod-nsHif1a retinas.

**Table S2: File type: xlsx**

Differentially upregulated genes in 661W cells after transfection with nsHif1a.

**Table S3: File type: xlsx**

Overlapping upregulated genes in rod-nsHif1a retinas and 661W cells after nsHif1a transfection.

**Table S4: File type: xlsx**

Gene Set Enrichment Analysis (GSEA) of differentially regulated genes in rod-*nsHif1a* retinas*.*

**Table S5: File type: xlsx**

Gene Ontology (GO) term 'Biological Processes' (BP) of differentially regulated genes in rod-*nsHif1a* retinas*.*

**Supplemental file 1: File type: png**

Original western blots, Figure 2B.
